# Association between weight-adjusted waist index and cardiovascular disease: a systematic review and meta-analysis

**DOI:** 10.3389/fendo.2025.1644035

**Published:** 2025-09-26

**Authors:** Kelly Pimenta, Edmore Madondo, Ayesha Mukhopadhyay, Jay Patel, Samuel Muhit, Fawaz Mzayek, Debra Bartelli, Matthew Smeltzer, Hongmei Zhang

**Affiliations:** ^1^ Division of Epidemiology, Biostatistics, and Environmental Health, School of Public Health, University of Memphis, Memphis, TN, United States; ^2^ Department of Global Pediatric Medicine, St. Jude Children's Research Hospital, Memphis, TN, United States; ^3^ John C Martin Center for Liver Research and Innovations, Kolkata, West Bengal, India; ^4^ TeamHealth, Edison, NJ, United States; ^5^ Hackensack Meridian Health, Edison, NJ, United States

**Keywords:** cardiovascular disease, weight-adjusted waist index, obesity, systematic review, meta-analysis

## Abstract

**Background:**

The increasing global prevalence of obesity and cardiovascular disease (CVD) represents a pressing public health challenge. Traditional obesity metrics, such as body mass index (BMI) and waist circumference (WC), have limitations in accurately predicting CVD risk. The weight-adjusted waist index (WWI), a novel metric combining WC and body weight, has been proposed as an alternative predictor of central obesity and its associated risks. This systematic review and meta-analysis aimed to evaluate the association between WWI and CVD.

**Methods:**

We conducted a systematic review of the literature in accordance with the Preferred Reporting Items for Systematic Reviews and Meta-Analyses (PRISMA) guidelines, and the study was registered with PROSPERO (ID CRD42024629861), searching PubMed, Scopus, and Google Scholar for observational studies examining the relationship between WWI and CVD. Data extraction and quality assessment were performed independently by two reviewers. A random-effects meta-analysis and subgroup analyzes were conducted to pool effect sizes, expressed as adjusted odds ratios (aORs) or adjusted hazard ratios (aHRs), and heterogeneity was evaluated using I², T², and Q statistics.

**Results:**

Ten studies comprising 170,297 participants were included. The pooled analysis revealed a significant positive association between WWI and increased CVD risk, with a pooled OR of 1.33 (95% CI: 1.17-1.48, *p* < 0.01). Moderate heterogeneity was observed (I² = 38.0%). Subgroup analyses showed stronger associations in studies conducted in the United States (OR: 1.35; 95% CI: 1.24 – 1.47) compared to China (OR: 1.32; 95% CI: 1.17 – 1.48). No significant differences were found between cross-sectional (OR: 1.33) and cohort studies (OR: 1.37).

**Conclusions:**

This study suggests a potential association between WWI and CVD, supporting its utility as an alternative measure of central obesity compared to traditional metrics. Despite these findings, moderate heterogeneity warrants further investigation into population-specific factors and mechanisms underlying the relationship between WWI and CVD. Future research should validate these findings across diverse populations and explore the clinical applications of WWI in CVD prevention strategies.

**Systematic review registration:**

https://www.crd.york.ac.uk/PROSPERO/, identifier CRD42024629861.

## Introduction

The escalating global prevalence of both obesity and cardiovascular disease (CVD) poses a pressing public health issue (1). The presence of obesity worldwide has doubled since the 1990s. By 2022, over 2.5 billion individuals were classified as overweight, of which 890 million were obese (2). Obesity is a multifactorial disease linked to biological, socioeconomic, and environmental factors which can contribute to adverse health outcomes (3–5). Obesity was previously most prevalent in high-income nations; however, in recent years, its prevalence has risen significantly in low-and-middle-income countries (LMICS). This increase has been attributed to factors such as economic expansion, the proliferation of street-food and fast-food options, and urbanization (6). Additionally, recent reports suggests that the rate of insufficient levels of physical activity in LMICS has also risen over the past 15 years (7). With this increase in obesity rates and its role as a prominent precursor, the risk of developing comorbidities, particularly CVD and related outcomes, escalates proportionally (8, 9).

Cardiovascular disease refers to general term for an incident that may cause damage to the heart muscle and blood vessels and includes hypertension, myocardial infarction, coronary artery disease, peripheral artery disease, aortic atherosclerosis, and cerebrovascular diseases, which include stroke and transient ischemic attack (10, 11). Despite efforts to improve CVD outcomes, the disease remains as one of the main causes of morbidity and mortality worldwide (12). In 2021, CVD was responsible for over 20 million deaths, an increase from 18 million deaths in 2000 (12, 13). Extensive and longitudinal investigations consistently reinforce the robust association between obesity and a markedly heightened risk of cardiovascular disease-related morbidity and mortality (7, 8). The findings emphasize the timely detection of obese individuals at an increased risk for CVD highlighting the role in the implementation of effective preventive interventions.

Body mass index (BMI), waist-to-height ratio (WtHR), and waist circumference (WC) stand as prevalent indices utilized in assessing obesity. However, previous investigations have shown that these indices do not provide an accurate measure of obesity. For instance, BMI does not differentiate between body fat and lean mass and has significant variation by sex and race/ethnicity (13). Additionally, WC does not distinguish between visceral abdominal fat and subcutaneous fat, and similarly, the WtHR, which is as simple as a ratio, can be challenging to interpret in clinical contexts (14–16). Considering these limitations, Park and colleagues proposed a novel obesity assessment metric in 2018, termed the weight-adjusted waist-index (WWI), which is calculated by dividing WC (in cm) by the square root of weight (in kg), where higher scores indicate higher levels of obesity (17). A significant advantage of WWI is its ability to integrate WC and individual weight, providing a more accurate presentation of central obesity (17). Central obesity is the presence of excess fat deposits in the abdominal region and is highly concerning due to its strong association with numerous adverse health outcomes (18). WWI has been shown to outperform traditional obesity indicators as predictors of chronic health conditions. For example, Wang et al. reported that WWI had a greater area under the curve (AUC) value (57.19%), compared to WC, weight (kg), and BMI (52.23%, 51.23%, and 49.96%, respectively), making it the most effective predictor of diabetic kidney disease (19). Similarly, Park et al., found WWI has a significant higher AUC value (0.736) than BMI, WC, and WHtR (0.597, 0.638, and 0.659, respectively) for predicting CVD, further establishing its utility as a predictor of chronic diseases (7). Central obesity is a well-established contributor to the development of CVD. It is characterized by excessive production and increased circulation of free fatty acids and triglycerides, which lead to lipid accumulation in the heart and other organs, ultimately contributing to hypertension and cardiovascular conditions. Moreover, central obesity is associated with reduced secretion of adiponectin, a hormone produced by white adipose tissue (WAT) that plays a crucial role in protecting the heart and reducing the risk of CVD (20) Subsequent research has shown that WWI demonstrates superior prognostic utility for various chronic health outcomes, including diabetic kidney disease, asthma, kidney stones, and depression when compared with traditional measures of obesity (21–24). These findings underscore the potential of WWI as a more scientifically strong predictor of certain CVDs.

The body of research examining the association between obesity- related indices and CVD is expanding, however findings remain inconsistent. Accumulating data has indicated that traditional indices such as BMI and WHtR are strongly associated with a rise in the prevalence of hypertension, stroke, and other CVDs (25). However, in recent years, controversy regarding the health outcomes due to overweight and obesity has grown in CVD patients, given findings of similar or lower all-cause mortality compared with their normal-weight counterparts. This is partly because these anthropometric measures do not clearly distinguish between muscle mass and fat mass. There is a need for a proper obesity index that accurately measures visceral fat by incorporating WC and taking body weight into consideration.

Despite growing interest, to the best of our knowledge, a systematic review on the association between WWI and CVD has not been performed. As the novel measure continues to be utilized, systematic reviews and meta-analyses are important tools to summarize the findings and assess overall statistical significance of the association with an increased power compared with that of individual studies. Therefore, the overall objective of this study was to review the available literature and undertake a systematic review and meta-analysis of the evidence to assess the magnitude of the association between WWI and CVD.

## Methods

### Data search strategy

**Figure 1:**
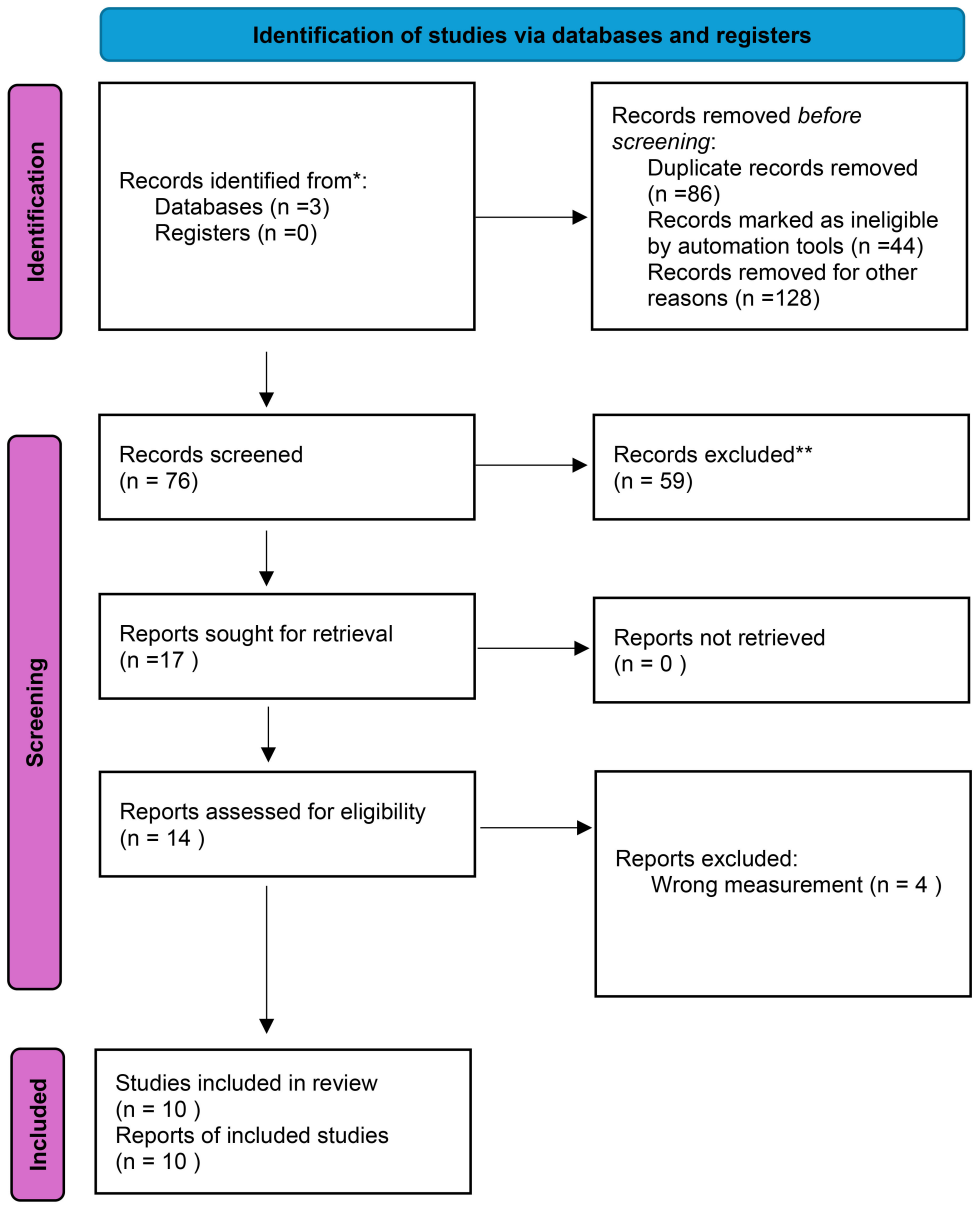
PRISMA flowchart for the systematic review and meta-analysis.

A systematic review of published studies was conducted in accordance with the Preferred Reporting Items for Systematic Reviews and Meta-Analysis (PRISMA) guidelines (**Figure 1**) (26). To explore the association between WWI and CVD, a comprehensive search was performed using PubMed Google Scholar, and Scopus. Search terms combining WWI and cardiovascular diseases were employed, with the following search string:

“(“Weight adjusted waist index” OR “WWI”) AND (“blood pressure” OR “hypertension” OR “cholesterol” OR “lipid” OR “lipids” OR “lipoprotein” OR “lipoproteins” OR “cardiovascular” OR “cardiovascular disease” OR “heart disease” OR “myocardial infarction” OR “ischemic heart disease”) AND (“observational” OR “cohort” OR “longitudinal”) AND (“human” OR “humans”)”. Keywords under the MeSH tree were included in the search strategy. The review was limited to studies published in, or translated into, English, with no restrictions on publication date to accommodate the relatively new terminology of WWI. The final search was conducted on November 12, 2024. Observational studies meeting the inclusion criteria were selected for analysis.

### Data extraction

Data extraction was independently carried out by two reviewers (K.P. and E.M.), ensuring a robust review process. Duplicates were removed, followed by an independent screening of titles, abstracts, and full texts by both reviewers. Studies that failed to meet the inclusion criteria were excluded (Appendix 1). Any discrepancies were resolved through detailed discussion and re-evaluation until a consensus was reached. Information, including author name, publication year, study location, sample size, and type of CVD measured, was extracted from each selected study. All included studies are listed in Appendix 2.

### Risk of bias assessment

Study quality was assessed independently by two reviewers (K.P. and E.M) using the National Institutes of Health quality assessment for observational cohort and cross-sectional studies which consists of 14 questions focusing on concepts that are key to a study’s internal validity: study population, sample size justification, exposure and outcome measurement, study statistical analyses and other assessments. Each study was scored as “Good”, “Fair” or “Poor” on the basis of the rater’s score. Additionally, we visually inspected a funnel plot to examine publication bias and assessed the symmetry of the plot using the Egger’s Regression Test (27).

### Meta-analysis

A random-effects meta-analysis was conducted to account for variations in study design, geographic location, and participant age. Heterogeneity among the included studies was assessed, and standardized effect sizes were calculated using adjusted odds ratios (aORs), adjusted hazard ratios (aHRs), and 95% confidence intervals (CIs) for cardiovascular outcomes. To ensure comparability across studies, all effect sizes were log-transformed. WWI was analyzed as a continuous measure in the studies. Heterogeneity was evaluated using Q, T2, and I2 statistics to determine its presence and magnitude. All the statistical analyses were performed using SAS version 9.4 (SAS Institute Inc.,Cary, NC, US) and R Studio, with statistical significance defined as *p* < 0.05.

### Subgroup analysis

The effects of different confounding factors, such as study design and study geographical location were tested by subgroup analysis. For the study methodology factors, study design was assessed (cross-sectional vs cohort). For demographic data, the study location (United States vs China) was compared. Pooled odds ratios (ORs) and 95% CI in each subgroup were calculated. The *X^2^* test was used to detect the heterogeneity between subgroups.

## Results

**Figure 1** shows the PRISMA flowchart of our systematic literature review, article screening, and study selection. Fifty-five (55) potential studies were retrieved. After excluding articles with inaccurate definitions of WWI or cardiovascular disease, and a lack of information, in total ten (10) studies were included in subsequent analyses (**Figure 1**).

The systematic review and meta-analysis included 10 studies (7 cross-sectional and 3 cohort studies) with a total of 170,297 participants. These studies were conducted exclusively in the United States (U.S.) and China, with the majority originating from the U.S. (70%). Overall, the included studies reported good study quality, except for Xie et al., which showed study quality as fair (**Table 1**). The aORs for the association between WWI and CVD ranged from 1.20 to 1.82 and the aHRs ranged from 1.17 to 1.95. The wide range of aORs and aHRs indicates variability in the strength of the association between WWI and cardiovascular events across the included studies. Standard errors (SEs) extracted from studies such as Ding et al. and Hu et al. indicate more precise estimates (0.07 and 0.06, respectively), whereas studies like Han et al. show larger uncertainty. All studies demonstrated statistically significant associations between WWI and CVD (**Table 1**). When reported, the mean age of the participants ranged from 47 to 69 years. In three studies where the mean age was not provided, the minimum age of participants was reported as 18 years with a maximum of over 70 years. The overall pooled odds ratio was 1.33 (95% CI: 1.25 – 1.42), indicating a statistically significant and positive association (p <0.01; **Table 2**); that is, the higher the WWI, the higher the odds of CVD.

**Table 1:** Characteristics of studies included.

STUDY ID	Author	Year	Country	Sample Size (N= 170,297)	Study Design	Outcome (Event)	Mean Age (Years)	Gender (Male, %)	Effect Size (aOR ^1^ [95% CI^3^])	SE^4^	Study Quality
1	Fang et al.	2023	US	21,040	Cross sectional	CHF, CHD, Angina, HA, Stroke	47.11	48.53	1.48[1.25,1.74]	0.13	Good
2	Ye et al.	2023	US	23,389	Cross sectional	Stroke	49.32	48.78	1.25[1.05,1.48]	0.11	Good
3	Zhang et al.	2022	US	25,509	Cross sectional	Heart Failure	46.77	48.84	1.20[1.04,1.38]	0.08	Good
4	Wang et al.	2023	US	39,156	Cross sectional	Hypertension	69.25	45.70	1.32[1.14,1.53]	0.10	Good
5	Xie et al.	2022	US	2,772	Cross sectional	Abdominal aortic calcification	57.72	48.29	1.82[1.20, 2.75]	0.40	Fair
6	Li et al.	2020	China	10,338	Prospective cohort	Hypertension	≥18 *	39.49	1.50[1.24, 1.82]	0.15	Good
7	Qin et al.	2022	US	3,802	Cross sectional	Abdominal aortic calcification	≥ 40 *	48.2	1.38[1.07, 1.78]	0.18	Good
									aHR^2^ [95% CI^3^]		
8	Ding et al.	2022	China	12,447	Prospective cohort	Cardiovascular mortality	59	40.6	1.17[1.05, 1.31]	0.07	Good
9	Han et al.	2023	US	26,882	Prospective cohort	Cardiovascular mortality	≥18*	49.23	1.95[1.30, 2.93]	0.42	Good
10	Hu et al.	2024	China	4,962	Retrospective cohort	Stroke	≥60*	51.9	1.30 [1.18, 1.42]	0.06	Good

^1^Adjusted Odds Ratio, ^2^Adjusted Hazard Ratio, ^3^Confidence Interval, ^4^Standard Error.

*Age Not Reported As Continuous, Minimum Age Provided.

CHF, Congestive Heart Failure.

CHD, Coronary Heart Disease.

HA, Heart Attack.

**Table 2:** Overall pooled summary and heterogeneity testing.

Pool Estimate		
OR^1^	*95% CI^2^*	*p*-value
1.33	1.25, 1.42	<0.01
Heterogeneity		
I^2^	95% CI^2^	
38.0%	0.0%-70.3%	
T^2^		
0.002	0.000, 0.089	
Q		*p-value*
14.52		0.10

^1^Odds Ratio

^2^Confidence Interval

### Heterogeneity testing

When the standardized variation between the observed effect sizes was tested, the Q value was 14.52 with an associated *p*-value of 0.10, indicating a moderate between-study variability. We further calculated T^2,^ which measures the variation of the underlying true effect size. Our *T^2^* value is 0.002, with a 95% CI (0.000-0.089), indicating a wide uncertainty in the variation of the underlying true effect sizes. To determine the degree of heterogeneity relative to the observed total dispersion, we calculated the *I* ^2^ statistic. We found *I* ^2^ = 38.0% with 95% CI (0.0%-70.3%), indicating moderate heterogeneity relative to the observed total dispersion (**Table 2**).

### Forest plot

**Figure 2:**
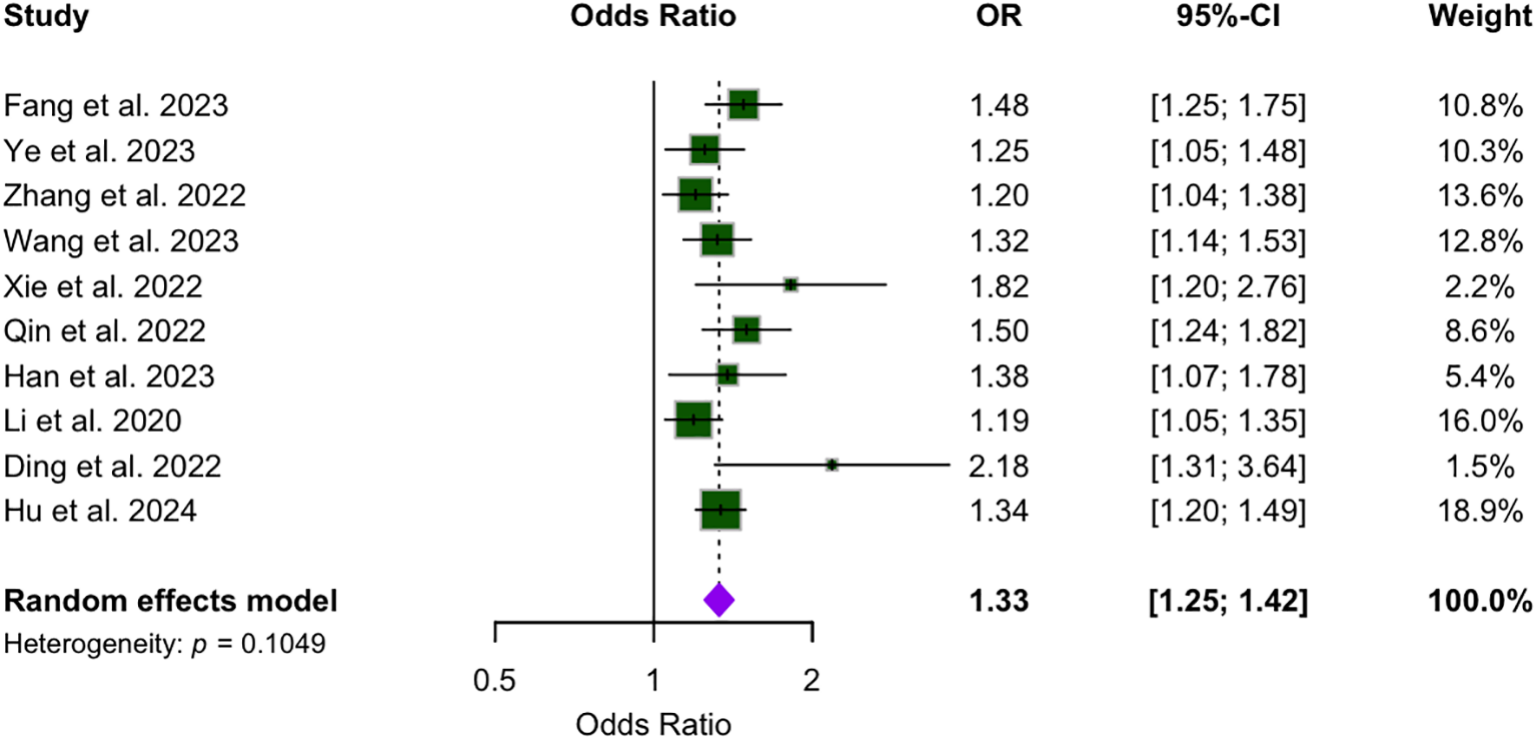
Forest plot of the association between WWI and cardiovascular disease.

**Figure 2** presents the analysis of the association between WWI and CVD across the included studies. The pooled effect estimate is 1.33, with a 95% CI ranging from 1.25 to 1.42. While most studies reported relatively narrow CIs, the publications by Han and Xie presented wider intervals. Notably, none of the ten studies reported a 95% CI that included 1, indicating a statistically significant association between WWI and CVD.

### Subgroup analysis

**Figure 3:**
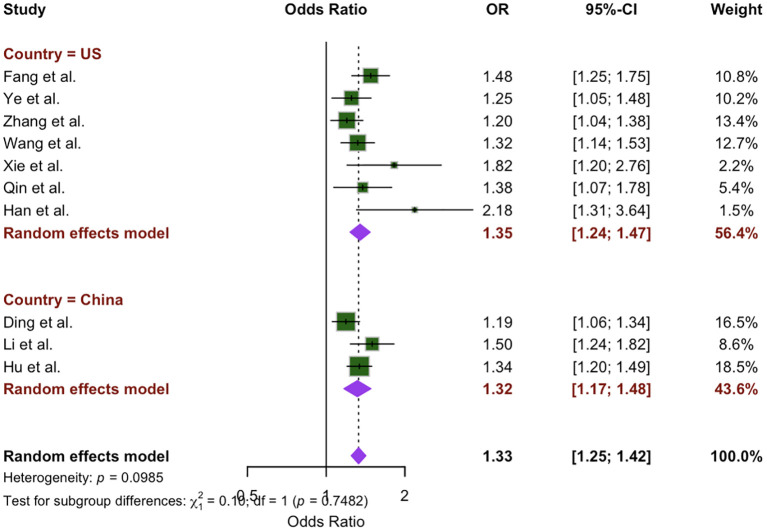
Location subgroup analysis of the association between WWI and CVD.

To evaluate the extent of heterogeneity in our study, we conducted a subgroup analysis based on differences identified by independent reviewers among the included studies. This analysis focused on regional variations, comparing studies conducted in the U.S. (7 studies) with those from China (3 studies). The meta-analysis indicated a greater OR for the U.S. population (OR: 1.35; 95% CI: 1.24 – 1.47, P = 0.12) compared to the Chinese population (OR: 1.32; 95% CI: 1.17 – 1.48, P = 0.10). However, the difference between location subgroups was not statistically significant (x^2^ = 0.10, *p* = 0.75), as shown in **Figure 3**.

**Figure 4:**
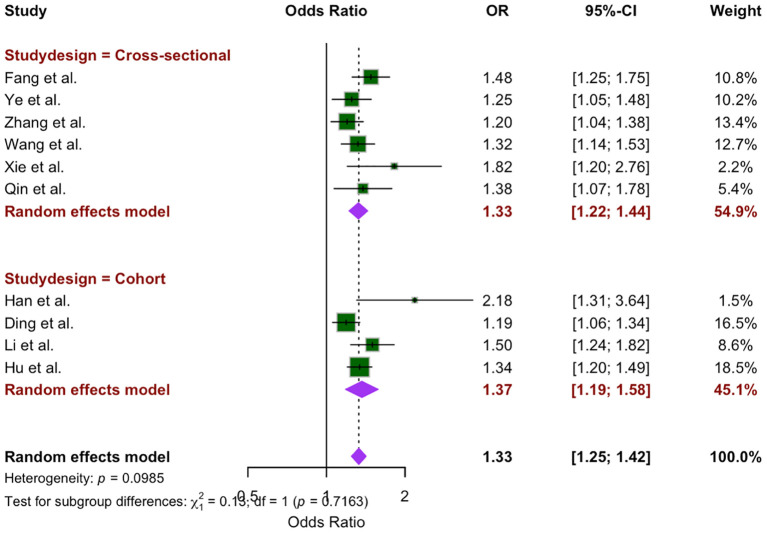
Study design subgroup analysis of the association between WWI and CVD.

Subgroup analysis was conducted on the basis of study design, comparing cross-sectional studies (6 studies) and cohort studies (4 studies). The OR for cross-sectional studies was 1.33 (95% CI: 1.22 –1.44, P = 0.27), whereas the OR for cohort studies was 1.37 (95% CI: 1.19 – 1.58, P = 0.04). The random-effects model revealed no statistically significant difference between the two study designs (x^2^ = 0.13, *p* = 0.72) (**Figure 4**).

**Figure 5:**
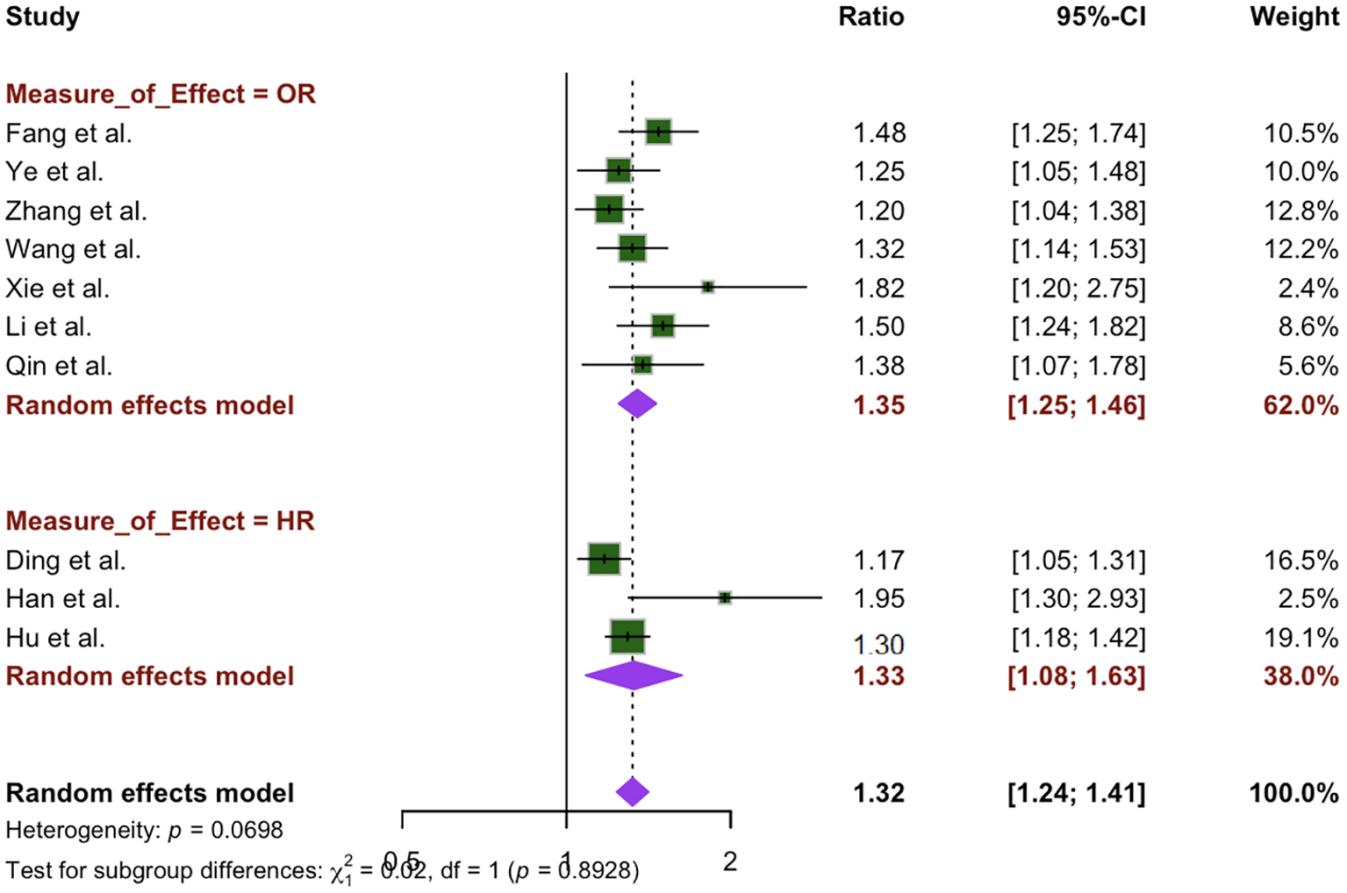
Measure of effect subgroup analysis of the association between WWI and CVD.

Due to the variation in OR and HR measure assumptions, a subgroup analysis was performed based on the measure of effect from each study, ORs (7 studies) and HRs (3 studies). The summary estimate of studies reporting ORs and studies reporting HRs are both statistically significant and similar, 1.35 (95% CI: 1.25 – 1.46) and 1.33 (95% CI: 1.08 – 1.63), respectively. The random-effects model revealed no statistically significant difference between the measures (x^2^ = 0.02, p=0.89. I^2^ was higher in the HR group than the OR group, showing greater variability, 68.7% and 24.1%, respectively. (**Figure 5**). Although no specific variables were identified as key drivers of the observed heterogeneity, differences in sample sizes and other unreported factors in the studies may have contributed to the variability.

### Publication bias

**Figure 6:**
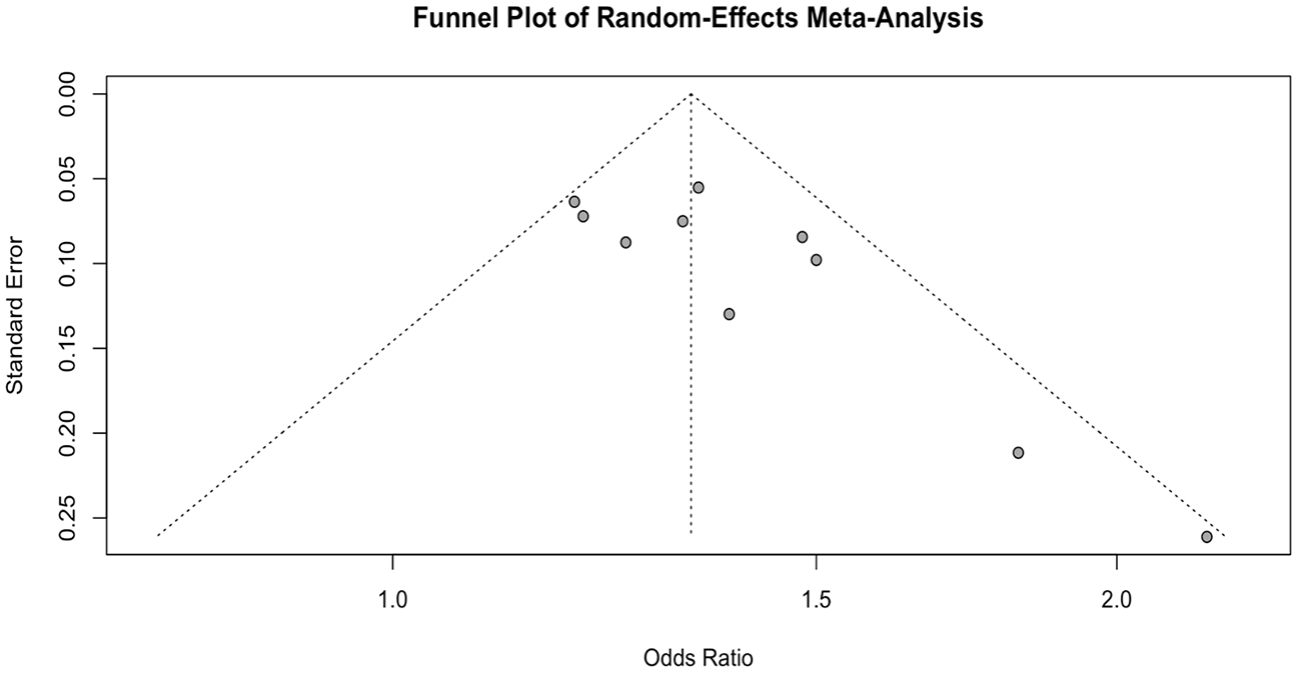
Funnel plot of the risk of bias in the meta-analysis.

Using Egger’s test and the visual of a funnel plot, the possibility of publication bias or the effects of small studies among the included studies was assessed (**Figure 6**). The funnel plot revealed an asymmetrical distribution, prompting the use of Egger’s regression test to examine the presence of publication bias. Moreover, Egger’s regression test indicated statistically significant asymmetry (P = 0.022), suggesting that the funnel plot asymmetry is unlikely due to random variation but attributable to publication bias.

## Discussion

To the best of our knowledge, this contemporary study is the first systematic review and meta-analysis to examine the association between WWI and CVD. The primary aim was to review the existing literature and conduct a meta-analysis to evaluate the consistency and magnitude of this association. Among the 10 included studies, most were non-male participants and were primarily from the US. A consistent association between WWI and CVD was observed across studies. The estimated overall weighted pooled effect (pooled OR = 1.33) demonstrates a harmful impact of WWI on CVD.

Our subgroup analysis results revealed the association between WWI and CVD is not statistically significantly irrespective of geographical location, with a larger summary estimate for the studies conducted in the US than those conducted in China. Key factors that may influence the different magnitudes of associations include differences in lifestyle and behavioral factors, differing healthcare access, and prevalence of obesity as the US has significantly higher rates of obesity and CVD factors compared to China (3). When looking at the subgroup analysis for study design, the overall random effect between cross-sectional and cohort studies varies slightly, potentially due to differences in study design, purpose, and methodology. Additionally, to better understand the various measures of association between WWI and CVD, we completed subgroup analysis based on ORs and HRs and found no statistical differences between subgroups allowing us to use the pooled estimate.

Given the increasing prevalence of overweight and obesity disorders nowadays, it is essential to assess obesity in an effective manner and then identify individuals at risk for CVD. Previous research examining the relationship between obesity and CVD has utilized indicators similar to WWI such as BMI, WtHR, and WC as indicators of obesity. Comparable to our findings, these studies consistently demonstrated that increased BMI, WtHR, and WC levels are significantly associated with an increased risk of CVD (28). For example, findings from the Women’s Health Study revealed that women with higher BMI levels had a 50% greater risk of stroke (hemorrhagic and ischemic) and 72% greater risk of ischemic stroke compared to those with lower BMI levels (29). Additionally, data from the Korean National Health Insurance Service program revealed a positive non-linear association between WC and all forms of CVD (p-trend <0.001) (30). Similarly, a study conducted in Mexico reported that 63.3% of individuals classified as obese based on WtHR were at increased risk for CVD, with 30.7% classified as being very high risk (31). However, these metrics are complex to measure and are susceptible to subjectivity.

Despite substantial evidence supporting a strong association between traditional obesity indicators and CVD, conflicting findings have emerged. These discrepancies are often described by the term “obesity paradox,” which refers to the observation that obese individuals may experience lower mortality risk compared to those of normal weight (32). This paradox suggests a non-linear association and potentially misleading message to the public, thus extrapolation of the findings needs to be cautious and subsequent studies to explore such complexity in associations is certainly warranted. For example, McAuley et al., in the Veterans Exercise Testing Study, reported a reduced mortality risk among overweight and obese males compared with normal-weight males (33). Similarly, studies have found lower mortality risk in older obese women with cancer than in their normal-weight counterparts (34). In stroke patients, a lower mortality risk was observed for individuals categorized as overweight (HR: 0.73, 95% CI: 0.66–0.81), obese (HR: 0.84, 95% CI: 0.73–0.98), or severely obese (HR: 0.84, 95% CI: 0.64-1.10), whereas underweight patients exhibited a significantly higher risk (HR: 1.63, 95% CI: 1.41-1.90) (35). These findings highlight the complex and sometimes counterintuitive relationship between obesity and health outcomes. The paradox may be partially explained by the limitations of traditional obesity indicators such as BMI, WC, and the WtHR, which fail to reliably differentiate between body fat and muscle mass (14). This limitation underscores the need to redefine obesity to improve the accuracy of its measurement.

WWI is a straightforward anthropometric measure calculated as WC (cm) divided by the square root of weight (kg) (cm/√kg). This index adjusts WC for weight using least-squares regression of log-transformed WC on log-transformed weight (17). Recent evidence suggests that WWI was positively associated with total and abdominal fat measures but inversely associated with appendicular skeletal muscle mass in older adults (36). Furthermore, recent evidence indicates that WWI is a better identificatory for CVD than are BMI, WC, and WHtR (25). Therefore, WWI may offer a comprehensive measure that combines the strengths of various anthropometric indicators, which further emphasizes the importance of our findings from meta-analyses regarding the connection between WWI and CVD.

As with any research, our study has significant strengths and limitations. A key strength is the large sample size, which increases the statistical power to detect significant differences. Furthermore, our aggregate results provide a more accurate estimate of the true effect size in the association between WWI and CVD. Subgroup analyses were also performed, allowing us to explore variations across different study designs and geographic locations. Additionally, the absence of time frame restrictions enabled a comprehensive assessment of the relationship between WWI and CVD. However, there are notable limitations. While our systematic review and meta-analysis did not restrict inclusion by geographic location, the findings are primarily based on data from Chinese and US populations, which may limit their generalizability and applicability to other regions. Although the two countries are geographically apart, they have comparable features; both countries span diverse socioeconomic and cultural lifestyles and each with a large population size. Thus, we feel that findings from these two countries have the potential to represent populations in other regions (37). Regardless, uncertainty remains regarding the generalizability of these results to broader populations. In addition, the high heterogeneity observed across the included studies likely stems from variability in study populations, designs, measurement methods, potential adjustments for confounders, as well as differences in geographic and temporal contexts. These factors can influence the strength and direction of the association, making it challenging to derive universal conclusions. Should such information become available, more granular subgroup analyses or meta-regression should be considered to strengthen our findings and enhance understanding of these associations. Thirdly, our meta-analyses exclusively utilized publications in three databases, PubMed, Google Scholar and Scopus, all of which are comprehensive and reputable with PubMed and Google Scholar a combined coverage of 85-98% in bioscience studies and Scopus 87-89% coverage in health science studies (38, 39). However, despite their comprehensive coverage, there remains a possibility that relevant publications available exclusively in other key databases such as Embase and Web of Science were not included in our study.

While the odds ratio suggests a linear association, further investigation is warranted to confirm whether the relationship is truly linear. For example, while a linear relationship may be suggested by the odds ratio, physiological responses (biological plausibility) to WWI and its effects on CVD risk are often complex. Non-linear patterns, such as a U-shaped relationship are commonly observed in similar anthropometric measures like BMI and waist circumference. Additionally, there can be additional residual confounding factors that were not adjusted for in the included studies. In the future, exploratory techniques, such as graphing predicted probabilities, testing non-linear associations using semi-parametric models, and conducting stratified analyses, can help identify potential deviations from linearity. Understanding whether the relationship is linear or non-linear is crucial for accurately interpreting the impact of WWI on CVD risk and for informing clinical or public health interventions. Additionally, to ensure that all summary estimates were comparable, we converted any HRs to ORs, although we recognize that each measure captures varying measures of risk and carry differing underlying assumptions, which can introduce bias to our estimates. Also, the Egger’s test- which is widely used to detect publication bias in meta-analyses- was statistically significant (p=0.0074), indicating potential publication bias. This is potentially due to the fact that more significant findings are more likely to be accepted for publication. Publication bias may weaken the validity and generalizability of the findings, despite the thoroughness of our search strategy. Finally, although CVD encompasses a wide range of conditions, our study focused on a limited subset due to the scarcity of studies investigating the associations between WWI and other types of CVD.

The findings from our meta-analyses support the importance of WWI as a risk factor of CVD. Integrating WWI into clinical and public health practice has potential to benefit clinicians and patients in CVS prevention and intervention. The assessment of WWI does not require advanced equipment and thus is feasible for implementation in almost all clinics or by health practitioners. We are confident that a more accurate indicator of obesity, e.g., WWI, could enhance risk stratification and support more effective care delivery. By synthesizing the available literature, we have gained a deeper understanding of the relationship between WWI and CVD.

## Conclusion

Our findings suggest that higher levels of WWI values may be associated with an increased risk of CVD. These results have important public health implications, as they can help inform clinical guidelines and shape health policy. Given the simplicity and accessibility of WWI measurement, it may serve as a practical tool for early identification of individuals at elevated cardiovascular risk. Further longitudinal and interventional studies are needed to confirm these associations and clarify underlying mechanisms.

## Data Availability

The original contributions presented in the study are included in the article/supplementary material. Further inquiries can be directed to the corresponding author/s.

## Appendix

### Appendix 1 Search strategy inclusion and exclusion criteria.

**Table:**

Criteria	Inclusion Criteria	Exclusion Criteria
Population	Studies involving patients with cardiovascular events.	Studies that did not evaluate any cardiovascular event.
Intervention/Exposure	Studies examining and reporting on the measure of weight-adjusted-waist index (WWI).	Studies that did not measure or report WWI.
Study Design	All observational studies.	Systematic review, Case reports, reviews, editorials, factsheets and commentaries.
Publication Type	All peer-reviewed journal articles and full-text articles.	All non-peer reviewed articles, Conference abstracts, posters.
Language	Article published in English.	Articles published in not English language.
Publication Date	Studies conducted from 2018-2024.	Studies conducted prior to 2018.

## Appendix 2 Included Studies

**Table:**

Study (first, author, year)	Reference
Fang et al., 2023	Fang, H., Xie, F., Li, K. et al. Association between weight-adjusted-waist index and risk of cardiovascular diseases in United States adults: a cross-sectional study. *BMC Cardiovasc Disord* 23, 435 (2023). https://doi.org/10.1186/s12872-023-03452-z.
Ye et al., 2023	Ye, J., Hu, Y., Chen, X. et al. Association between the weight-adjusted waist index and stroke: a cross-sectional study. *BMC Public Health* **23**, 1689 (2023). https://doi.org/10.1186/s12889-023-16621-8.
Zhang et al., 2022	Zhang D, Shi W, Ding Z, Park J, Wu S, Zhang J. Association between weight-adjusted-waist index and heart failure: Results from National Health and Nutrition Examination Survey 1999-2018. Front Cardiovasc Med. 2022 Dec 14;9:1069146. doi: 10.3389/fcvm.2022.1069146. PMID: 36588556; PMCID: PMC9794568.
Wang et al., 2023	Wang J, Yang QY, Chai DJ, Su Y, Jin QZ, Wang JH. The relationship between obesity associated weight-adjusted waist index and the prevalence of hypertension in US adults aged ≥60 years: a brief report. Front Public Health. 2023 Oct 6;11:1210669. doi: 10.3389/fpubh.2023.1210669. PMID: 37869197; PMCID: PMC10587597.
Xie et al., 2022	Xie F, Xiao Y, Li X, Wu Y. Association between the weight-adjusted-waist index and abdominal aortic calcification in United States adults: Results from the national health and nutrition examination survey 2013-2014. Front Cardiovasc Med. 2022 Sep 14;9:948194. doi: 10.3389/fcvm.2022.948194. PMID: 36186965; PMCID: PMC9515490.
Qin et al., 2022	Qin, Z., Du, D., Li, Y. et al. The association between weight-adjusted-waist index and abdominal aortic calcification in adults aged ≥ 40 years: results from NHANES 2013–2014. *Sci Rep* **12**, 20354 (2022). https://doi.org/10.1038/s41598-022-24756-8.
Li et al., 2020	Li Q, Qie R, Qin P, Zhang D, Guo C, Zhou Q, Tian G, Liu D, Chen X, Liu L, Liu F, Cheng C, Han M, Huang S, Wu X, Zhao Y, Ren Y, Zhang M, Hu D, Lu J. Association of weight-adjusted-waist index with incident hypertension: The Rural Chinese Cohort Study. Nutr Metab Cardiovasc Dis. 2020 Sep 24;30(10):1732-1741. doi: 10.1016/j.numecd.2020.05.033. Epub 2020 Jun 7. PMID: 32624344.
Ding et al., 2022	Ding, Congcong & Shi, Yumeng & Li, Junpei & Li, Minghui & Hu, Lihua & Rao, Jingan & Liu, Liang & Zhao, Peixu & Xie, Chong & Zhan, Biming & Zhou, Wei & Wang, Tao & Zhu, Lingjuan & Huang, Xiao & Bao, Huihui & Cheng, Xiaoshu. (2022). Association of weight-adjusted-waist index with all-cause and cardiovascular mortality in China: A prospective cohort study. Nutrition, Metabolism and Cardiovascular Diseases. 32. 10.1016/j.numecd.2022.01.033.
Han et al., 2023	Han Y, Shi J, Gao P, Zhang L, Niu X, Fu N. The weight-adjusted-waist index predicts all-cause and cardiovascular mortality in general US adults. Clinics (Sao Paulo). 2023 Jul 11;78:100248. doi: 10.1016/j.clinsp.2023.100248. PMID: 37441866; PMCID: PMC10362289.
